# Mean Platelet Volume and Platelet Distribution Width as Markers in the Diagnosis of Acute Gangrenous Appendicitis

**DOI:** 10.1155/2015/542013

**Published:** 2015-11-25

**Authors:** Zhe Fan, Jiyong Pan, Yingyi Zhang, Ziyi Wang, Ming Zhu, Baoshun Yang, Lei Shi, Huirong Jing

**Affiliations:** ^1^Department of General Surgery, The Third People's Hospital of Dalian, Non-Directly Affiliated Hospital of Dalian Medical University, Dalian 116033, China; ^2^Department of General Surgery, The Second Hospital of Dalian Medical University, Dalian 116023, China; ^3^Department of General Surgery, The Second Hospital of Dalian Medical University, Northern Yard, Dalian 116023, China

## Abstract

*Introduction.* Acute gangrenous appendicitis (AGA) is a common medical condition; however, the grade of appendicitis usually cannot be established preoperatively. We have attempted to identify some indicators, such as the mean platelet volume (MPV) and the platelet distribution width (PDW), to diagnose AGA.* Aims.* To evaluate whether or not the MPV and PDW are suitable markers to diagnose AGA.* Methods.* A retrospective study of 160 patients with AGA and 160 healthy patients was undertaken. Disease diagnosis was confirmed based on the pathologic examination of surgical specimens. Patient white blood cell (WBC) count, neutrophil ratio (NR), platelet (PLT) count, MPV, PDW, and hematocrit (HCT) were analyzed. Receiver operating characteristic (ROC) curves were used to evaluate the sensitivity and specificity of these indices in AGA.* Results.* There were no significant differences between the AGA and control groups in age and gender. Compared to the control group, the WBC count, NR, and PDW were significantly higher (*P* < 0.001, resp.) and the MPV and HCT were significantly lower (*P* < 0.001, resp.) in the AGA group. The diagnostic specificities of the WBC count, NR, PLT count, MPV, PDW, and HCT were 86.3%, 92.5%, 58.1%, 81.7%, 83.9%, and 66.3%, respectively. Therefore, the NR had the highest diagnostic specificity for the diagnosis of AGA.* Conclusions.* This is the first study to assess the MPV and PDW in patients with AGA. Our present study showed that the MPV is reduced and the PDW is increased in patients with AGA; the sensitivity of PDW was superior to the MPV. A decreased MPV value and an increased PDW could serve as two markers to diagnose AGA. The NR had the highest specificity for the diagnosis of AGA.

## 1. Introduction

Acute appendicitis is a common medical condition encountered in general surgery. There are three grades to illustrate acute appendicitis (focal appendicitis, suppurative appendicitis, and gangrenous appendicitis) [1, 2]. The clinical diagnosis of acute appendicitis is often based on medical history, physical examination, blood tests, and iconographic examination [3]. Timely emergency surgery should be carried out to avoid peritonitis, especially in patients with suppurative or gangrenous appendicitis, before perforation occurs [1]; however, the grade of appendicitis cannot usually be evaluated based on the above examinations.

Platelets (PLTs) have effects on haemostasis and regulate inflammatory events. PLTs are more highly activated when inflammatory mediators are released [4]. The mean platelet volume (MPV) and platelet distribution width (PDW) are two PLT parameters of the complete blood count [5]. After PLT production increases, MPV changes accordingly [4–6]. It has been reported that the MPV is affected in patients with acute pancreatitis, ulcerative colitis, rheumatoid arthritis, and ankylosing spondylitis [4, 6, 7].

In the present study, we focused on the relationship between MPV and PDW and acute gangrenous appendicitis (AGA). Dinc et al. [8] suggested the PDW as a new index in the diagnosis of acute appendicitis, and we verified changes in the PDW in patients with AGA.

## 2. Materials and Methods

One hundred and sixty patients with AGA and 160 healthy people were enrolled in our study. AGA was diagnosed based on postoperative pathologic examination. The patients in the AGA group were treated at The Third People's Hospital of Dalian between June 2011 and June 2014 and healthy patients (control group) with normal physical examinations were enrolled from our physical examination center during the same period. This retrospective study was approved by the hospital ethics committee.

Patients with the following conditions were excluded from the study: <15 years of age; alcohol consumption; cigarette smoking; diabetes mellitus; hypertension; morbid obesity; and severe comorbidities (heart failure, peripheral vascular disease, hematologic disorders, acute or chronic infections, cancer, and hepatic disease) [9, 10].

All blood samples were collected into tubes containing EDTA (potassium ethylenediaminetetraacetate) through the cephalic vein and assayed using internationally certified devices. All results were available in <10 min [8].

The white blood cell (WBC) count, NR, PLT count, MPV, PDW, and hematocrit (HCT) were collected from AGA and control groups.

The reference values were 4–10 × 10^9^/L for WBC, 40%–70% for NR, 100–300 × 10^9^/L for PLT, 7.6–13.2 fL for the MPV, 12%–16.5% for the PDW, and 40%–50% for the HCT.

### 2.1. Statistical Analysis

Research data were analyzed using SPSS 20.0 software (SPSS for Windows; SPSS, Inc., Chicago, IL, USA). The continuous data are presented as the mean ± standard deviation (SD), Student's *t*-test was used for the comparison between the two groups, and a *χ* ^2^ test was used for comparing count data between the two groups. A normal distribution was analyzed using binary logistic analysis. Logistic analysis and receiver-operating curve (ROC) analysis were used to describe the parameters in the AGA and control groups. The results were examined within the 95% CI, and a *P* < 0.05 was considered statistically significant.

## 3. Results

The mean ages of the patients were 45.6 ± 19.6 years (range, 14–89 years) in the AGA group and 43.0 ± 12.5 years (range, 15–87 years) in the control group. There were no significant differences between the AGA and control groups with respect to age and gender (Table 1).

The mean WBC counts in the AGA and control groups were 13.06 ± 4.64 × 10^9^/L (range, 1.81–25.66 × 10^9^/L) and 6.12 ± 1.62 × 10^9^/L (range, 3.26–11.49 × 10^9^/L; *P* < 0.001).

NR in the AGA and control groups was 84.21 ± 9.34% (range, 43.3–97.24%) and 54.57 ± 8.11% (range, 32.62–75.71%; *P* < 0.001).

The PLT counts in the AGA and control groups were 210.29 ± 60.41 × 10^9^/L (range, 90–428 × 10^9^/L) and 220.09 ± 45.28 × 10^9^/L (range, 105–348 × 10^9^/L; *P* = 0.102 > 0.05).

The MPV in the AGA and control groups were 9.21 ± 1.38 × 10^9^/L (range, 6.6–12.9 × 10^9^/L) and 10.91 ± 2.72 × 10^9^/L (range, 8.82–43.6 × 10^9^/L; *P* < 0.001).

The PDW in the AGA and control groups was 15.25 ± 1.90 × 10^9^/L (range, 9.9–18.1 × 10^9^/L) and 12.50 ± 1.93 × 10^9^/L (range, 1.3–19.1 × 10^9^/L; *P* < 0.001).

The HCT in the AGA and control groups was 40.93 ± 5.48% (range, 24.82–52.5%) and 43.92 ± 3.77% (range, 33.4–52.4%; *P* < 0.001) (Table 2).

Based on the results in Table 2, the WBC counts, NR, and PDW were significantly increased (*P* < 0.001) and the MPV and HCT were significantly decreased (*P* < 0.001) in the AGA group compared to the control group. There were no significant changes in PLT count (*P* > 0.05).

Binary logistic analysis was carried out and the results were as follows: MPV (*P* = 0.000); PDW (*P* = 0.000); PLT (*P* = 0.012); HCT (*P* = 0.001); neutrophil ratio (NR) (*P* = 0.026); and WBC (*P* = 0.024) (*P* < 0.05, resp.).

**Figure 1:**
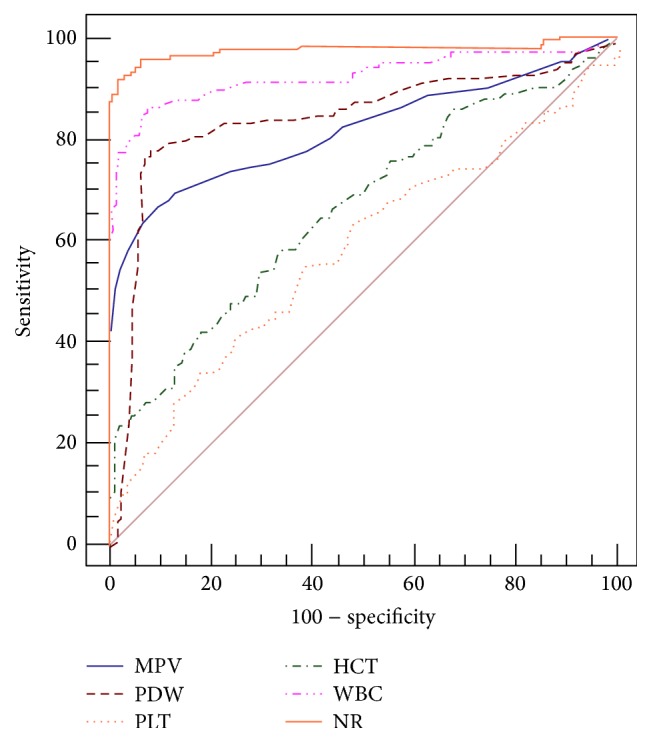
ROC curves for MPV, PDW, PLT count, HCT, NR, and WBC count.

The ROC curves were analyzed for the following indices, as shown in Figure 1:

Area under curve (AUC): MPV (0.817), PDW (0.839), PLT count (0.581), HCT (0.663), NR (0.975), and WBC count (0.923);

Cut-off value: MPV (9.6× 10^9^/L), PDW (15.1× 10^9^/L), PLT count (179× 10^9^/L), HCT (40.3 %), NR (69.5 %), and WBC count (8.45× 10^9^/L);

Sensitivity and specificity: [MPV (66.25% and 91.19%), PDW (76.3% and 93.1%), PLT count (33.13% and 83.02%), HCT (41.88% and 82.39%), NR (92.5% and 96.9%), and WBC count (86.3% and 92.5%)] (Table 3).

## 4. Discussion

Acute appendicitis often presents as an acute abdomen. In recent studies [9, 11, 12], researchers considered the MPV to be helpful in diagnosing acute appendicitis; however, opinions have not been consistent. Previous studies [8, 9, 11] have concluded that acute appendicitis can induce changes in the MPV or PDW; however, the conclusions drawn in the two studies about MPV were not consistent. Perhaps there is another reason to account for the discrepant results, such as ethnicity and geographic influence. Erdem et al. [9] reported that the MPV was markedly lower in acute appendicitis groups compared to control groups. In contrast, Narci et al. [11] concluded that the MPV was significantly higher in acute appendicitis groups compared with control groups. Uyanik et al. [12] concluded that the MPV has no diagnostic value with respect to acute appendicitis. Based on our analysis of the recent literature, we suggest that there is no relationship between the MPV and acute appendicitis. Classic symptoms and physical examination findings could be used to diagnose acute appendicitis; however, it is difficult to distinguish gangrenous cases from several types of appendicitis. Therefore, we focused on the MPV in the diagnosis of AGA. A MEDLINE search for articles in the English language from 1981 to 2014 with the terms “MPV”/“PDW” and “acute gangrenous appendicitis” revealed no entries.

MPV has been studied as an inflammatory marker in several diseases. MPV represents an index of PLT function. An increase in “young” platelets and an aggregation of large platelets could lead to higher MPV values. PLT size and activity are influenced by cytokines, such as IL-3 or IL-6 [11]. In many chronic diseases, the MPV increases, while in many acute diseases the MPV decreases [9]. Specifically, the MPV decreases in patients with ulcerative colitis, rheumatoid arthritis, and ankylosing spondylitis [8, 13, 14], and the MPV increases in patients with ankylosing spondylitis, familial Mediterranean fever, Behcet's disease, and psoriasis [15, 16]. In the current study, the MPV was markedly decreased in patients with AGA, in agreement with Erdem et al. [9]. In our research, the sensitivity of MPV in the current study was slightly lower than that of PDW, which indicates that the MPV could be as a diagnostic marker for acute appendicitis or AGA. The PDW can represent the heterogeneity of thrombocyte volume [10]. There are two studies [10, 17] which illustrate the correlation between the PDW and acute appendicitis; both studies demonstrated an increase in the PDW. Our study was the third study to show an increase in the PDW in patients with acute appendicitis and to show an increase in the PDW in patients with AGA.

In the present study, a decrease in the HCT was shown in patients with AGA. This is the first study to show a relationship between HCT and AGA. A MEDLINE search for “hematocrit” and “inflammation” revealed no entries. Indeed, alternate mechanisms may exist and additional research should be conducted.

The increases in the WBC counts and neutrophil-to-lymphocyte ratio have been used for the diagnosis of acute appendicitis [1, 9, 18]. Similarly, our research showed that the NR has the best sensitivity (92.5%); the sensitivities of the WBC count and PDW were 86.3% and 76.3%, respectively.

Based on the work of Albayrak et al. [10] and our results herein, the PDW may be a new marker to diagnose acute appendicitis. In the present study, the PDW increased in patients with AGA. A new diagnostic algorithm for the diagnosis of AGA may include the WBC count, NR, and PDW.

Although some factors have been excluded from the study, there were some limitations, including the small sample size, and the onset of acute appendicitis was not recorded.

## 5. Conclusion

Based on the current study, the MPV is a new index for diagnosing AGA. While the MPV was clearly lower in patients with AGA, the MPV did not have a higher sensitivity compared with the PDW. We confirmed that the PDW is a new, highly sensitive parameter with which we diagnose AGA. The WBC count and NR have high sensitivity in diagnosing AGA.

## Figures and Tables

**Table 1:** Demographic data for patients with AGA and control groups.

Characteristic	Control group	AGA group	*P* value
Age	43.0 ± 12.5 years	45.6 ± 19.6 years	0.155
Gender (male/female)	99/61	102/58	0.644

**Table 2:** Laboratory results for patients with AGA and control groups.

Indexes	Control group	AGA group	*P* value
WBC	6.12 ± 1.62 × 10^9^/L	13.06 ± 4.64 × 10^9^/L	0.000
NR	54.57 ± 8.11%	84.21 ± 9.34%	0.000
PLT	220.09 ± 45.28 × 10^9^/L	210.29 ± 60.41 × 10^9^/L	0.102
MPV	10.91 ± 2.72 × 10^9^/L	9.21 ± 1.38 × 10^9^/L	0.000
PDW	12.50 ± 1.93 × 10^9^/L	15.25 ± 1.90 × 10^9^/L	0.000
HCT	43.92 ± 3.77%	40.93 ± 5.48%	0.000

**Table 3:** ROC curves/cut-off values/sensitivity/specificity with AGA and control groups.

Indexes	AUC	Cut-off value	Sensitivity	Specificity
WBC	0.923	8.45 × 10^9^/L	86.3%	92.5%
NG	0.975	69.5%	92.5%	96.9%
PLT	0.581	179 × 10^9^/L	33.13%	83.02%
MPV	0.817	9.6 × 10^9^/L	66.25%	91.19%
PDW	0.839	15.1 × 10^9^/L	76.3%	93.1%
HCT	0.663	40.3%	41.88%	82.39%

## Abbreviations

MPV: Mean platelet volume
PDW: Platelet distribution width
PLT: Platelet count
NR: Neutrophil ratio.
